# 97. The Utilization of Echocardiography in Children with *Staphylococcus aureus* Bacteremia

**DOI:** 10.1093/ofid/ofad500.013

**Published:** 2023-11-27

**Authors:** Richard Bui, Jesus G Vallejo, Sheldon L Kaplan, Jonathon C McNeil

**Affiliations:** Baylor College of Medicine, Houston, TX; Baylor College of Medicine, Houston, TX; Baylor College of Medicine, Houston, TX; Baylor College of Medicine, Houston, TX

## Abstract

**Background:**

Among children with *Staphylococcus aureus* bacteremia (SAB) the most common focus of infection is musculoskeletal with infective endocarditis (IE) being relatively rare. Echocardiography (“echo”) is routinely performed in adults with SAB with rates of IE as high as 25%. We evaluated the utility of echo in the setting of SAB in children at a tertiary children’s hospital.

**Methods:**

Children (< 18 years-old) with at least one blood culture positive for *S. aureus* from 2019-2021 were identified from an ongoing surveillance study at Texas Children’s Hospital in Houston, TX. Medical records were reviewed for all subjects. For this study, we aimed to define the frequency and clinical correlates of a) the utilization of echo and b) evidence of echo-positive IE. Echo evidence of IE was based on the Duke criteria.

**Results:**

331 cases of SAB were identified in children with a median age of 4.9 years. Medical comorbidities were present in 58% of subjects. Musculoskeletal infections were the most common diagnoses followed by CLABSI (**Figure 1**). 22.1% of infections were due to MRSA. The median duration of bacteremia was 1 day (IQR: 1-2). Echo was performed in 140 (42.3%) subjects. Those who underwent echo had a higher degree of medical complexity, particularly congenital heart disease (CHD, **Figure 2**), and had a longer duration of bacteremia (median 2 vs. 1 day, p=0.01). Four subjects underwent transesophageal echo. Nine subjects met Duke echo major criteria for IE (6.4%, **Figure 3**). Subjects with echo-positive IE were more likely to have CHD than those without (66.7% vs. 20.6%, p=0.006); duration of bacteremia or fever among those with and without echo positive IE were not significantly different. Among subjects with CHD and SAB, 15.6% had echo diagnosed IE. 4/9 subjects with vegetations had a single day of SAB.

**Figure:**
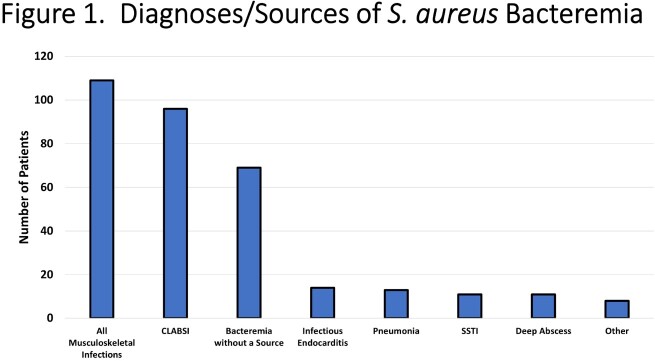

**Figure:**
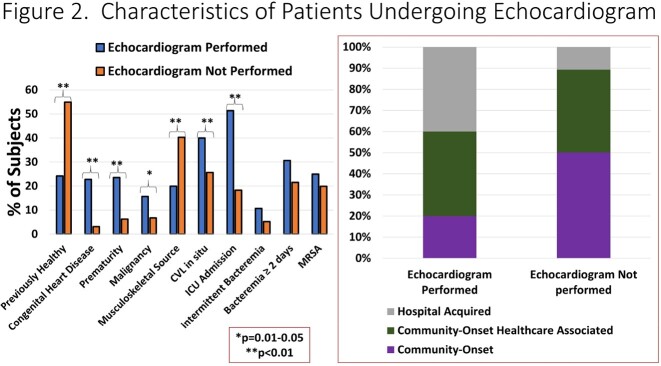

Figure 2A. Depicts select clinical characteristics with respect to their frequency among subjects who did or did not undergo echocardiogram. Figure 2B. Demonstrates the relative proportion of cases with community-associated, community-onset healthcare-associated and hospital-associated infection.

**Figure:**
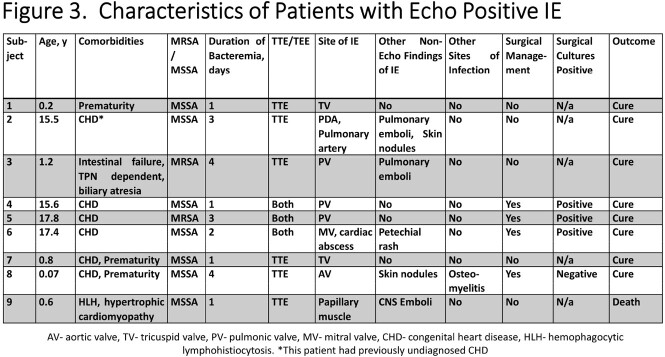

The figure illustrates select clinical characteristics for subjects with echocardiogram confirmed infective endocarditis (IE)

**Conclusion:**

While a large proportion of children with SAB at our center underwent echo, the frequency of IE by imaging was approximately 6%. Children with CHD are at high risk for IE, however, identifying other children who would benefit the most from echo in the setting of SAB is challenging. Notably, even with short durations of SAB, IE was documented in children with comorbidities. Further multicenter work with very large sample sizes is needed to optimize echo use in children with SAB.

**Disclosures:**

**Sheldon L. Kaplan, MD**, MeMed: Grant/Research Support|Pfizer: Grant/Research Support|Pfizer: Honoraria **Jonathon C. McNeil, MD**, Allergan: Grant/Research Support|Nabriva Therapeutics: Grant/Research Support

